# Principles of high–fidelity, high–density 3–d neural recording

**DOI:** 10.1186/1471-2202-15-S1-P122

**Published:** 2014-07-21

**Authors:** Caroline Moore-Kochlacs, Jorg Scholvin, Justin P Kinney, Jacob G Bernstein, Young Gyu Yoon, Scott K Arfin, Nancy Kopell, Ed S Boyden

**Affiliations:** 1 Graduate Program for Neuroscience, Boston University, Boston, MA 02215, USA; 2 McGovern Institute, Massachusetts Institute of Technology, MA 02139, USA; 3 Department of Mathematics and Statistics, Boston University, Boston, MA 02215, USA; 4 Media Lab, Massachusetts Institute of Technology, MA 02139, USA; 5 Electrical Engineering and Computer Science, Massachusetts Institute of Technology, MA 02139, USA; 6 Brain and Cognitive Science, Massachusetts Institute of Technology, MA 02139, USA; 7 Biological Engineering, Massachusetts Institute of Technology, MA 02139, USA

## 

New probe technologies, neural amplifier systems, and data acquisition systems enable the extracellular electrical recording of ever greater numbers of neurons in the live mammalian brain. These recordings have the potential to increase our understanding of neuronal network dynamics, but much remains uncharacterized about the possibilities and limitations of extracellular techniques. We explore these possibilities and limitations in the context of spike sorting and probe design. Spike sorting is a critical analysis step for extracellular data, which attempts to separate raw electrode traces into the activity patterns of individual neurons. Given the labor associated with manual spike sorting of large datasets, the necessity for automated spike sorting method will only increase. An automated method would ideally commit no errors in spike assignment – that is, it would associate each extracted individual neuron with all the spikes fired by a single neuron, and with no spikes not fired by that same neuron. The elimination of errors would reduce the reliance on manual validation, saving large amounts of analysis time, and also reduce downstream biases in data analyses introduced by errors in spike sorting. We explore designs for multi-electrode probes and spike sorting methods that in combination allow high accuracy in spike assignment with many neurons extracted. To this end, first, we ask if an automated spike sorting method with zero spike assignment errors is possible. Second, we explore what multi-electrode probe designs produce optimal yield using this method.

We have constructed a spike sorting method for the case of spatially dense high channel count extracellular recordings, first applying a well-established source separation technique called Independent Components Analysis (ICA) to continuous recordings. Second, we apply a classifier to the ICA components, keeping only putative single neuron units that are well separated from noise and other units. To test this algorithm, we simulated multielectrode probe data that encompasses many of the realistic variations and noisinesses of natural neural data, including spatial non-linearities in spike shape from individual cells. Running this algorithm against this simulated data, for a wide range of classifier parameters, we find that no spike assignment errors are committed. This result is robust to changes in neural firing rate, neural density, Gaussian noise, and increasing electrode density on the probes.

However, for probes with electrode counts similar to those in commercial probes (10-50), only a handful of neurons are extracted. Exploring the space of probe designs, we find that designing probes with higher electrode density (for a fixed area) can compensate for this low yield. As the electrode density on the probe increases, the number of neurons extracted increases to some saturation. We also find that as the electrode density increases, we are able to extract neurons with spike peak magnitudes below the thresholding noise floor.

Thus, the construction of very high density multielectrode arrays, coupled to the algorithm here proposed, may yield experimental approaches for recording very large numbers of neurons in the live brain, and automatically analyzing the resulting spike trains.

